# Serum Granulin Concentrations Are Elevated in Prediabetes and Newly Diagnosed Diabetes: A Cross-Sectional Study

**DOI:** 10.3390/jcm14186566

**Published:** 2025-09-18

**Authors:** Yu-Hsuan Chou, Yuan Kao, Ka Chon Chan, Hsuan-Wen Chou, Yu-Cheng Liang, Hung-Tsung Wu, Horng-Yih Ou

**Affiliations:** 1 Department of Internal Medicine, National Cheng Kung University Hospital, College of Medicine, National Cheng Kung University, Tainan 704, Taiwan; 2 Department of Emergency Medicine, Chi Mei Medical Center, Tainan 710, Taiwan; 3 Department of Internal Medicine, School of Medicine, College of Medicine, National Cheng Kung University, Tainan 701, Taiwan

**Keywords:** biomarker, diabetes, granulin, obesity, prediabetes

## Abstract

**Background**: Recently, the incidence of diabetes has increased. A rapid method for the diagnosis of prediabetes is needed. Although granulin levels are associated with obesity and insulin resistance, it remains unclear whether serum granulin concentration may serve as a biomarker of prediabetes and diabetes. Here, we examined the association between serum granulin and glycemic status in a clinical population. **Methods:** In total, 180 age- and sex-matched participants with normal glucose tolerance (NGT), impaired fasting glucose (IFG), impaired glucose tolerance (IGT) and newly diagnosed diabetes (NDD) were recruited. Serum granulin levels were measured via an enzyme-linked immunosorbent assay. Multivariate linear regression analysis was performed to evaluate the relationships between the level of granulin and different glycemic statuses. The utility of the granulin concentration for diagnosis of prediabetes and diabetes was evaluated with a receiver operating characteristic (ROC) curve. **Results:** Serum granulin concentrations were significantly greater in the IFG, IGT and NDD groups than in the NGT group. Multiple linear regression analysis revealed that obesity and glycemic status were independently associated with granulin concentrations. The ROC curve analysis revealed an area under the curve of 0.781 (95% CI, 0.709–0.853; *p* < 0.001). **Conclusions:** An elevated serum granulin concentration has potential utility as a biomarker for screening prediabetes and diabetes.

## 1. Introduction

Type 2 diabetes mellitus (T2DM) is a highly prevalent global health condition that contributes to diverse and significant health complications [1]. It is closely linked to an elevated risk of cardiovascular disorders, metabolic dysfunction-associated fatty liver disease (MAFLD), and multiple malignancies [2,3,4,5,6]. Furthermore, prediabetes has the risk of progression to T2DM, and individuals with prediabetes also have a high burden of cardiovascular risk [7]. Therefore, early detection and screening prediabetes are also important, and rapid and reliable methods for early detection are urgently needed.

Currently, the oral glucose tolerance test (OGTT) and measurement of glycated hemoglobin (HbA1c) are the gold standards for prediabetes screening [8]. However, the OGTT is cumbersome because of the need for multiple blood draws, while HbA1c may not have sufficient sensitivity for screening for prediabetes [9]. Furthermore, the HbA1c test can be unreliable in conditions with red blood cell turnover or abnormal hemoglobin binding of glucose, including hemoglobinopathies, anemia, iron deficiency and renal impairment [10,11]. Therefore, novel biomarkers for prediabetes screening must be both sensitive and specific.

Progranulin, the precursor of the glycoprotein granulin, has been identified as an adipokine closely linked to obesity, insulin resistance, and type 2 diabetes (T2DM) [12,13]. Animal studies have further demonstrated its role in mediating high-fat diet-induced insulin resistance [14] and obesity [15]. Mechanistically, progranulin has been shown to induce adipose tissue insulin resistance and autophagic imbalance via tumor necrosis factor receptor 1 [16,17]. A key study also revealed that progranulin deficiency can prevent diet-induced obesity by inhibiting inflammation in both the hypothalamus and adipose tissue [18]. Nevertheless, clinical evidence regarding the relationship between granulin and glycemic status remains limited, and it is unclear whether serum granulin concentrations are consistently altered in individuals with prediabetes and diabetes.

In the present study, we investigated the relationships between granulin levels and different glycemic statuses in a clinical population. The participants had either normal glucose tolerance (NGT), impaired fasting glucose (IFG), impaired glucose tolerance (IGT), or newly diagnosed diabetes (NDD). By comparing the serum granulin concentrations between groups, we sought to determine whether this measurement could serve as a potential biomarker for prediabetes screening.

## 2. Materials and Methods

### 2.1. Study Population

The participants in this case–control study were screened between February 2019 and July 2022 following a routine physical examination. All patient samples were deidentified and the subjects provided informed consent prior to their participation. Participants were eligible for inclusion if they were adults aged 18 years or older and provided informed consent to participate in the study. Individuals were excluded if they had a history of alcohol consumption exceeding 20 g per day within the previous year; tested positive for hepatitis B surface antigen or hepatitis C antibody; had laboratory evidence of other liver diseases, including autoimmune hepatitis, primary biliary cholangitis, Wilson’s disease, hemochromatosis, or other hepatic malignancies; had a serum creatinine level greater than 1.5 mg/dL; were pregnant; or had a diagnosis of advanced malignant disease.

A total of 180 participants were enrolled and classified into four groups: normal glucose tolerance (NGT, *n* = 47), impaired fasting glucose (IFG, *n* = 45), impaired glucose tolerance (IGT, *n* = 48), and newly diagnosed diabetes (NDD, *n* = 40).

Each participant was evaluated with a standard 75 g oral glucose tolerance test (OGTT) according to the criteria of the American Diabetes Association. On the basis of the test results, patients were grouped as follows: NGT if fasting plasma glucose (FPG) was <5.6 mmol/L and the 2 h plasma glucose was <7.8 mmol/L without a history of diabetes; IFG if FPG was 5.6–6.9 mmol/L and the 2 h plasma glucose was <7.8 mmol/L; IGT if FPG was <5.6 mmol/L and the 2 h plasma glucose was 7.8–11.1 mmol/L; and NDD if FPG was ≥7.0 mmol/L or the 2 h plasma glucose ≥11.1 mmol/L [8].

### 2.2. Clinical Variables

Body mass index (BMI, kg/m^2^) was calculated from body height and weight. Obesity was defined as a BMI ≥ 27.0, according to the criteria defined by the Health Promotion Administration, Ministry of Health and Welfare in Taiwan [19].

Blood pressure was measured following a conventional attended office protocol. The participants were seated and rested for at least five minutes before measurement. Two consecutive readings were taken, and the mean of the two readings was used for subsequent analyses. Hypertension was defined as a systolic blood pressure (SBP) > 130 mmHg or diastolic blood pressure (DBP) > 80 mmHg [20]. After an overnight 12 h fasting period, all the subjects were subjected to regular biochemical blood tests, including fasting plasma glucose (FPG), lipid profile, hepatic transaminase [alanine aminotransferase (ALT) and aspartate aminotransferase], and creatinine. These tests were performed by the Department of Laboratory Medicine. Serum total cholesterol, triglyceride (TG), and high-density lipoprotein cholesterol (HDL-C) levels were measured using a Hitachi 747E auto-analyzer (Tokyo, Japan). The low-density lipoprotein cholesterol (LDL-C) levels were calculated by the Friedewald formula. Dyslipidemia was defined as the presence of any of the following: total cholesterol > 200 mg/dL, triglycerides > 150 mg/dL, low-density lipoprotein cholesterol (LDL-C) > 100 mg/dL, or high-density lipoprotein cholesterol (HDL-C) < 40 mg/dL in men or <50 mg/dL in women [21].

Plasma concentrations of high-sensitivity (hs)-CRP were measured using commercial enzyme-linked immunosorbent assay (ELISA) assay kits (intra-assay CV < 2.9%, inter-assay CV < 4.7%; Immunology Consultants Laboratory, Newberg, OR, USA). Serum insulin was measured with an ultrasensitive ELISA (Mercodia, Uppsala, Sweden). Insulin resistance was determined by the homeostasis model assessment-insulin resistance (HOMA-IR) index as fasting insulin (μU/mL) × FPG (mmol/L)/22.5 [22].

The serum granulin concentration was measured by commercial ELISA kits (intra-assay CV = 5.21%, inter-assay CV = 4.95%; FineTestBiotech, Wuhan, China).

### 2.3. Statistical Analysis

All the statistical analyses were conducted using SPSS software, version 27.0. Normally distributed continuous variables are presented as mean ± standard deviation (SD). Chi-squared tests were used for comparisons of categorical variables, whereas one-way analysis of variance (ANOVA) was applied to assess differences in continuous variables across groups. Scheffe’s method was used for post hoc analysis to identify significant differences between groups. Multiple linear regression analysis was performed to determine the factors independently associated with granulin levels. The beta coefficient and 95% confidence intervals (CIs) are reported as standardized estimates. Multiple comparisons were performed to assess the independent associations between variables and granulin levels. Statistical significance was defined as *p* < 0.05. Receiver operating characteristic (ROC) curve analysis was performed to evaluate whether granulin level can be used to discriminate NGT from prediabetes and diabetes. The area under the curve (AUC) was calculated to quantify the discriminative ability of the diagnostic test. The 95% confidence intervals of sensitivity and specificity were calculated via the Wilson method.

## 3. Results

One-hundred-and-eighty participants were classified into four groups. The baseline characteristics of the study population are summarized in Table 1, which shows significant differences between the groups in fasting plasma glucose (*p* < 0.001), creatinine (*p* = 0.015), alanine aminotransferase (*p* = 0.042), hs-CRP (*p* = 0.023), HDL-cholesterol (*p* = 0.027), and HOMA-IR (*p* = 0.025).

The NDD group showed significantly higher levels of fasting plasma glucose, hs-CRP, and HOMA-IR compared to the other groups. Conversely, HDL was highest in the NGT group, while the IFG group had the highest creatinine levels.

**Figure 1:**
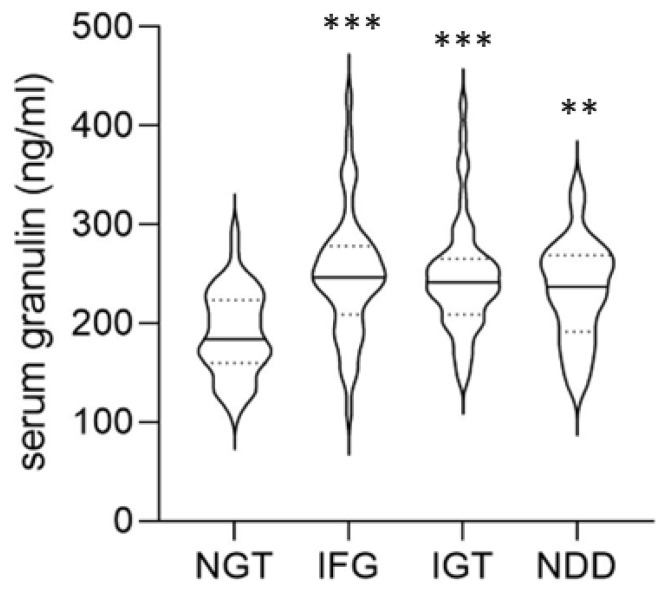
Plasma granulin concentrations are increased in individuals with prediabetes or diabetes. Violin plot of plasma granulin concentrations in participants with normal glucose tolerance (NGT; *n* = 47), impaired fasting glucose (IFG; *n* = 45), impaired glucose tolerance (IGT; *n* = 48) and newly diagnosed diabetes (NDD; *n* = 40). The plot illustrates the probability density of the data at different levels, along with the median (solid line) and interquartile range (dashed lines). ** *p* < 0.01 and *** *p* < 0.001 as compared with the NGT group.

Serum granulin concentrations were significantly greater in the IFG (*p* < 0.001), IGT (*p* < 0.001) and NDD (*p* = 0.005) groups, than in the NGT group. However, no statistically significant differences were found between the IFG and IGT (*p* = 0.998). There was also no statistically significant difference between the IFG and NDD groups (*p* = 0.534), or IGT and NDD groups (*p* = 0.636) (Figure 1).

To further elucidate the independent factors associated with serum granulin concentrations, we conducted a multivariate linear regression analysis. A consistent model was obtained using both stepwise and backward selection strategies, which is presented in Table 2. In model 1, We found that obesity was independently associated with granulin concentrations (β = 29.68, [95% CI = 1.49, 57.87]; *p* < 0.05) after adjusting for confounding factors, including sex, age, ALT, creatinine, hypertension, dyslipidemia, and the hs-CRP level. Additionally, glycemic status was independently associated with granulin concentrations (comparing NGT with IFG, IGT and NDD; β = 48.78, [95% CI = 30.98, 66.58]; *p* < 0.001) as shown in model 2. In model 3, we assessed the independent effect of glycemic status on plasma granulin concentrations. We identified independent associations between granulin concentrations and IFG (β = 55.92, 95% CI = 32.10–79.74; *p* < 0.001), IGT (β = 56.01, 95% CI = 34.99–77.04; *p* < 0.001), and NDD (β = 34.00, 95% CI = 11.61–56.40; *p* = 0.003), with NGT serving as the reference group.

**Figure 2:**
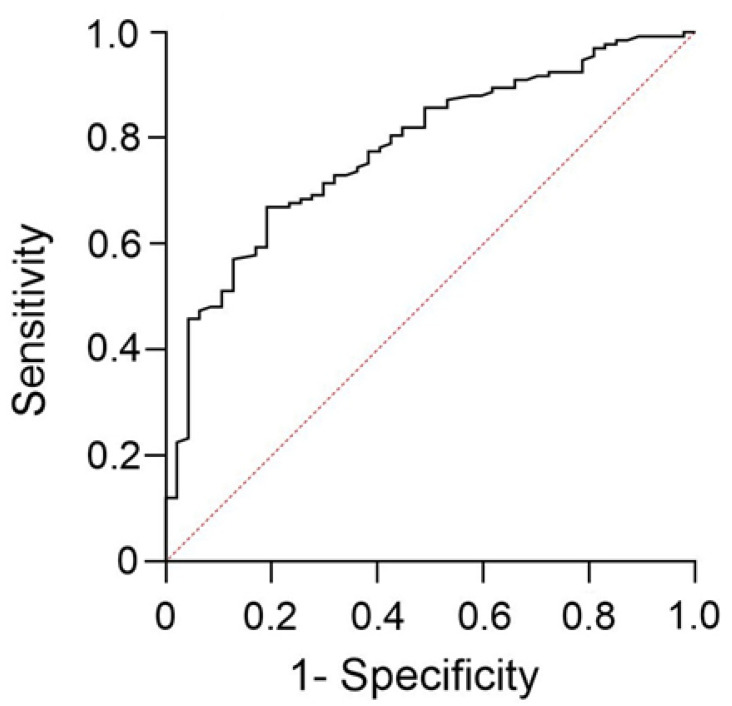
Receiver operating characteristic curve for predicting impaired fasting glucose, impaired glucose tolerance and diabetes. An optimal serum granulin cut-off value of 218.09 ng/mL yielded an AUC of 0.781 (95% CI = 0.709–0.853; *p* < 0.001) for predicting prediabetes and mellitus, with a sensitivity of 71.4% (95% CI = 63.2–85.3%) and a specificity of 70.2% (95% CI = 55.9–81.4%).

The analysis of the ROC curve revealed a statistically significant correlation between granulin levels and participant glycemic status (prediabetes and newly diagnosed diabetes) (Figure 2). The AUC was 0.781 (95% CI = 0.709–0.853; *p* < 0.001). An optimal serum granulin cutoff value of 218.09 ng/mL was used to identify individuals with prediabetes with a sensitivity of 71.4% (95% CI = 63.2–85.3%), and specificity of 70.2% (95% CI = 55.9–81.4%).

## 4. Discussion

In this study, our results revealed a previously undescribed relationship between granulin levels and glycemic status in individuals with normal glucose tolerance, prediabetes and diabetes. Compared with those in controls, granulin levels were significantly elevated in individuals with prediabetes and diabetes. In the multiple linear regression analysis, both obesity (β = 29.68, [95% CI = 1.49, 57.87]; *p* < 0.05) and glycemic status (β = 48.78, [95% CI = 30.98, 66.58]; *p* < 0.001) were independently associated with granulin concentrations. ROC curve analysis revealed an AUC of 0.781, indicating good predictive ability. We also selected a cutoff value of 218.09 ng/mL, achieving a sensitivity of 71.4% and a specificity of 70.2%. These findings suggest that elevated serum granulin concentrations may have potential utility as biomarkers for prediabetes screening.

In our method design, we adopted the Taiwan-specific BMI criteria for obesity (BMI ≥ 27.0) instead of the WHO guideline. This was to better reflect the unique characteristics of the local population, as this criterion is more sensitive for identifying obesity-related risks in Taiwan. We initially included BMI as a continuous variable in a multiple regression analysis. However, the association with our outcome reached a borderline statistical significance (*p* = 0.057), suggesting a non-linear or weak relationship. Therefore, to better evaluate obesity’s potential confounding effect on our results, we decided to categorize participants based on their obesity status in our subsequent analyses. Additionally, while we reported the exact *p*-values for all statistical tests in the tables, we used the notation *p* < 0.001 for extremely small values (e.g., 2 × 10^−6^) to ensure clarity and readability.

Early detection of prediabetes is a crucial public health concern given the increasing prevalence of diabetes and its associated complications. Although HbA1c is a widely used and convenient diagnostic tool, there is no consensus on an optimal cutoff value for prediabetes screening [23]. OGTTs are more strongly correlated with insulin resistance and β-cell function than HbA1c [24], but their clinical applications are limited by the need for multiple blood draws. Recently, several novel biomarkers, particularly inflammatory markers, have been proposed as potential tools for prediabetes screening [25,26]. However, an accurate and practical method for the early detection of glycemic dysregulation is still lacking. Furthermore, confounding factors such as lipid metabolism and the presence of MAFLD may affect test performance [27,28]. Against this background, our finding that serum granulin levels reflect glycemic status may have important clinical implications, especially for the early identification of individuals at risk for diabetes. These results also suggest a complementary role for granulin, potentially serving as a marker of adipose tissue dysfunction or inflammation.

Previous studies have shown that progranulin is associated with inflammatory responses and plays a role in the development of T2DM and obesity [29,30]. It may also aggravate insulin resistance by activating autophagy [31]. Moreover, progranulin levels are elevated in individuals with T2DM compared with nondiabetic controls [32], and elevated progranulin levels were observed in obese individuals with predominant visceral fat accumulation [30,33]. The progranulin concentration also appears to be closely linked to glucose metabolism, as it is associated with the HbA1c level and 2 h post-challenge plasma glucose level [30,32,33]. Moreover, higher progranulin levels are observed in individuals with advanced diabetic microvascular complications, including diabetic nephropathy and diabetic retinopathy [34,35,36]. Serum progranulin levels are significantly associated with inflammatory markers, including C-reactive protein and interleukin-6, and have also been shown to be positively correlated with components of metabolic syndrome [29]. Furthermore, alterations in the concentrations of progranulin and other adipokines, including chemerin, were observed in diabetic patients following insulin therapy. This observation suggests a link between these adipokine levels and the patient’s glycemic status [37]. On the basis of these studies, it is reasonable to expect that serum granulin levels may reliably reflect prediabetes and diabetes status.

Our data showed that obesity was independently associated with granulin concentration after adjustment for confounding factors, and that granulin levels were significantly associated with glycemic status. These findings are in line with the idea that granulin may contribute to the development of obesity and diabetes through inflammatory mechanisms. Given the role of progranulin and granulin in the inflammatory cascade, our findings suggest that granulin may mediate inflammation-induced metabolic dysfunction.

This study has several limitations. First, its cross-sectional design precludes causal inference. Although we observed an association between serum granulin and glycemic status, it remains uncertain whether elevated granulin contributes to diabetes pathogenesis or merely reflects metabolic dysregulation. Longitudinal studies are needed to clarify this relationship. Second, generalizability may be limited, as participants were recruited from a single medical center in Taiwan and consisted exclusively of an Asian population. Recruitment through a health screening program may also introduce selection bias. Validation in larger, multicenter cohorts with diverse populations is therefore required. Third, residual confounding from unmeasured factors cannot be excluded. Detailed dietary patterns, physical activity, socioeconomic status, and genetic predispositions were not assessed and may have influenced the observed associations. Finally, future in vitro and in vivo studies are needed to elucidate the physiological role of granulin and define its clinical value in risk stratification or disease monitoring.

## 5. Conclusions

This study demonstrated that serum granulin levels are significantly elevated in individuals with IFG, IGT, and diabetes, suggesting its potential as a novel biomarker for prediabetes screening. A serum granulin cutoff value of 218.09 ng/mL was identified, providing a sensitivity of 71.4% and a specificity of 70.2% for detecting prediabetes.

## Figures and Tables

**Table 1:** Clinical characteristics of the study participants in each group.

Characteristic	NGT	IFG	IGT	NDD	*p*
*n*	47	45	48	40	--
Age (years)	61.0 ± 11.0	62.1 ± 12.8	62.7 ± 12.0	61.9 ± 12.1	0.928
Sex (F/M)	18/29	17/28	19/29	15/25	0.997
BMI (kg/m^2^)	22.9 ± 2.8	23.8 ± 3.5	23.8 ± 3.2	23.5 ± 2.8	0.455
SBP (mmHg)	124.1 ± 17.2	127.3 ± 16.3	127.8 ± 16.1	128.6 ± 20.3	0.639
DBP (mmHg)	72.6 ± 9.4	73.0 ± 11.3	73.2 ± 9.8	75.3 ± 11.0	0.643
ALT (U/L)	23.4 ± 9.7	31.3 ± 24.6	21.8 ± 8.6	26.8 ± 20.4	0.042
AST (U/L)	25.9 ± 6.5	27.8 ± 16.7	23.8 ± 4.9	26.3 ± 11.7	0.351
Creatinine (μmol/L)	75.80 ± 15.17	85.65 ± 21.88	79.38 ± 17.35	73.59 ± 18.82	0.015
hs-CRP (mg/L)	2.9 ± 4.5	4.4 ± 7.3	2.9 ± 4.6	6.7 ± 9.4	0.023
Total cholesterol (mmol/L)	5.27 ± 1.04	5.26 ± 0.93	5.24 ± 0.99	5.41 ± 1.25	0.882
Triglyceride (mmol/L)	1.10 ± 0.45	1.27 ± 0.64	1.31 ± 0.73	1.46 ± 0.75	0.086
HDL (mmol/L)	1.53 ± 0.43	1.34 ± 0.40	1.34 ± 0.39	1.32 ± 0.28	0.027
LDL (mmol/L)	3.23 ± 0.99	3.34 ± 0.78	3.29 ± 0.90	3.42 ± 1.04	0.820
FPG (mmol/L)	4.79 ± 0.40	5.83 ± 0.27	4.84 ± 0.55	7.17 ± 3.42	<0.001
HOMA-IR	0.46 ± 0.31	0.68 ± 0.37	0.55 ± 0.44	0.78 ± 0.79	0.025

Data are expressed as the mean ± standard deviation. ALT, alanine aminotransferase; AST, aspartate aminotransferase; BMI, body mass index; DBP, diastolic blood pressure; FPG, fasting plasma glucose; HDL, high-density lipoprotein; LDL, low-density lipoprotein; HOMA-IR, homeostatic model assessment for insulin resistance; hs-CRP, high-sensitivity C-reactive protein; SBP, systolic blood pressure.

**Table 2:** Regression analysis of plasma granulin concentrations and clinical variables.

Variable	Model 1				Model 2				Model 3			
	β (95% CI)	Standard β	*p*-Value	Adjusted R Square	β (95% CI)	Standard β	*p*-Value	Adjusted R Square	β (95% CI)	Standard β	*p*-Value	Adjusted R Square
Age	−0.592 (−1.455, 0.271)	−0.186	0.177	0.042	−0.519 (−1.313, 0.275)	−0.107	0.199	0.190	−0.566 (−1.358, 0.225)	−0.117	0.159	0.203
sex	−21.623 (−45.056, 1.809)	−0.122	0.070		−18.131 (−39.717, 3.454)	−0.156	0.099		−15.915 (−37.481, 5.652)	−0.137	0.147	
ALT	0.394 (−0.127, 0.916)	0.119	0.138		0.343 (−0.137, 0.823)	0.104	0.160		0.350 (−0.131, 0.832)	0.106	0.152	
Creatinine	0.559 (−0.088, 1.205)	0.175	0.090		0.425 (−0.172, 1.022)	0.133	0.161		0.308 (−0.295, 0.912)	0.097	0.314	
Hypertension	5.711 (−12.934, 24.357)	0.605	0.545		2.836 (−14.342, 20.014)	0.025	0.745		4.434 (−12.685, 21.563)	0.039	0.610	
hs-CRP	0.651 (−0.766, 2.068)	0.907	0.366		0.352 (−0.956, 1.659)	0.041	0.596		0.642 (−0.686, 1.970)	0.074	0.341	
Dyslipidemia	−10.995 (−36.940, 14.949)	−0.837	0.404		−6.903 (−30.808, 17.001)	−0.043	0.569		−6.944 (−30.690, 16.801)	−0.043	0.564	
Obesity	29.680 (1.494, 57.865)	0.164	0.039		21.624 (−4.461, 47.710)	0.119	0.104		20.689 (−5.232, 46.609)	0.114	0.117	
Glycemic status	--		--		48.779 (30.980, 66.578)	0.390	<0.001		--		--	
IFG vs. NGT	--		--		--		--		55.922 (32.102, 79.743)	0.387	<0.001	
IGT vs. NGT	--		--		--		--		56.012 (34.986, 77.039)	0.447	<0.001	
NDD vs. NGT	--		--		--		--		34.000 (11.605, 56.395)	0.256	0.003	

Dependent variable: serum granulin concentrations; ALT, alanine aminotransferase; IFG, impaired fasting glucose, NGT, normal glucose tolerance; IGT, impaired glucose tolerance; NDD, newly diagnosed diabetes.

## Data Availability

To protect patient privacy and confidentiality. The dataset supporting our conclusions contains sensitive clinical information derived from patient records at our institution. In accordance with the ethical approval granted by our Institutional Review Board (IRB) and the terms of patient consent, we are obligated to maintain strict control over the data to prevent any potential risk of patient re-identification.

## References

[1] Hossain M.J., Al-Mamun M., Islam M.R. (2024). Diabetes mellitus, the fastest growing global public health concern: Early detection should be focused. Health Sci. Rep..

[2] Hafidh K., Malek R., Al-Rubeaan K., Kok A., Bayram F., Echtay A., Rajadhyaksha V., Hadaoui A. (2022). Prevalence and risk factors of vascular complications in type 2 diabetes mellitus: Results from discover Middle East and Africa cohort. Front. Endocrinol..

[3] Lu Y., Wang W., Liu J., Xie M., Liu Q., Li S. (2023). Vascular complications of diabetes: A narrative review. Medicine.

[4] Scott E.S., Januszewski A.S., O’cOnnell R., Fulcher G., Scott R., Kesaniemi A., Wu L., Colagiuri S., Keech A., Jenkins A.J. (2020). Long-Term Glycemic Variability and Vascular Complications in Type 2 Diabetes: Post Hoc Analysis of the FIELD Study. J. Clin. Endocrinol. Metab..

[5] Lawler T., Hibler E., Walts Z.L., Giurini L., Steinwandel M., Lipworth L., Murff H.J., Zheng W., Andersen S.W. (2024). Associations of diabetes and mortality among colorectal cancer patients from the Southern Community Cohort Study. Br. J. Cancer.

[6] Jung I., Koo D.J., Lee W.Y. (2024). Insulin Resistance, Non-Alcoholic Fatty Liver Disease and Type 2 Diabetes Mellitus: Clinical and Experimental Perspective. Diabetes Metab. J..

[7] Echouffo-Tcheugui J.B., Selvin E. (2021). Prediabetes and What It Means: The Epidemiological Evidence. Annu. Rev. Public. Health.

[8] American Diabetes Association Professional Practice Committee, ElSayed N.A., McCoy R.G., Aleppo G., Balapattabi K., Beverly E.A., Early K.B., Bruemmer D., Ebekozien O., Echouffo-Tcheugui J.B. (2024). 2. Diagnosis and Classification of Diabetes: Standards of Care in Diabetes—2025. Diabetes Care.

[9] Barry E., Roberts S., Oke J., Vijayaraghavan S., Normansell R., Greenhalgh T. (2017). Efficacy and effectiveness of screen and treat policies in prevention of type 2 diabetes: Systematic review and meta-analysis of screening tests and interventions. BMJ.

[10] Lundholm M.D., Emanuele M.A., Ashraf A., Nadeem S. (2020). Applications and pitfalls of hemoglobin A1C and alternative methods of glycemic monitoring. J. Diabetes Complicat..

[11] Yadav N., Kumar Mandal A. (2023). Interference of hemoglobin variants in HbA(1c) quantification. Clin. Chim. Acta.

[12] Eriksen J.L., Mackenzie I.R. (2008). Progranulin: Normal function and role in neurodegeneration. J. Neurochem..

[13] Korolczuk A., Bełtowski J. (2017). Progranulin, a New Adipokine at the Crossroads of Metabolic Syndrome, Diabetes, Dyslipidemia and Hypertension. Curr. Pharm. Des..

[14] Matsubara T., Mita A., Minami K., Hosooka T., Kitazawa S., Takahashi K., Tamori Y., Yokoi N., Watanabe M., Matsuo E. (2012). PGRN is a key adipokine mediating high fat diet-induced insulin resistance and obesity through IL-6 in adipose tissue. Cell Metab..

[15] Park C.B., Lee C.H., Cho K.W., Shin S., Jang W.H., Byeon J., Oh Y.R., Kim S.J., Park J.W., Kang G.M. (2024). Extracellular Cleavage of Microglia-Derived Progranulin Promotes Diet-Induced Obesity. Diabetes.

[16] Zhou B., Li H., Liu J., Xu L., Guo Q., Sun H., Wu S. (2015). Progranulin induces adipose insulin resistance and autophagic imbalance via TNFR1 in mice. J. Mol. Endocrinol..

[17] Li H., Zhou B., Xu L., Liu J., Zang W., Wu S., Sun H. (2014). Circulating PGRN is significantly associated with systemic insulin sensitivity and autophagic activity in metabolic syndrome. Endocrinology.

[18] Lee C.H., Park C.B., Kim H.K., Jang W.H., Min S.H., Kim J.B., Kim M.S. (2025). Macrophage-Specific Progranulin Deficiency Prevents Diet-Induced Obesity through the Inhibition of Hypothalamic and Adipose Tissue Inflammation. Diabetes Metab. J..

[19] Chu N.F. (2005). Prevalence of obesity in Taiwan. Obes. Rev..

[20] Whelton P.K., Carey R.M., Aronow W.S., Casey D.E., Collins K.J., Dennison Himmelfarb C., DePalma S.M., Gidding S., Jamerson K.A., Jones D.W. (2018). 2017 ACC/AHA/AAPA/ABC/ACPM/AGS/APhA/ASH/ASPC/NMA/PCNA Guideline for the Prevention, Detection, Evaluation, and Management of High Blood Pressure in Adults: Executive Summary: A Report of the American College of Cardiology/American Heart Association Task Force on Clinical Practice Guidelines. Hypertension.

[21] Pappan N., Awosika A.O., Rehman A. (2025). Dyslipidemia. StatPearls.

[22] Matthews D.R., Hosker J.P., Rudenski A.S., Naylor B.A., Treacher D.F., Turner R.C. (1985). Homeostasis model assessment: Insulin resistance and β-cell function from fasting plasma glucose and insulin concentrations in man. Diabetologia.

[23] Zhou X., Pang Z., Gao W., Wang S., Zhang L., Ning F., Qiao Q. (2010). Performance of an A1C and fasting capillary blood glucose test for screening newly diagnosed diabetes and pre-diabetes defined by an oral glucose tolerance test in Qingdao, China. Diabetes Care.

[24] Lorenzo C., Wagenknecht L.E., Hanley A.J., Rewers M.J., Karter A.J., Haffner S.M. (2010). A1C between 5.7 and 6.4% as a marker for identifying pre-diabetes, insulin sensitivity and secretion, and cardiovascular risk factors: The Insulin Resistance Atherosclerosis Study (IRAS). Diabetes Care.

[25] Dorcely B., Katz K., Jagannathan R., Chiang S.S., Oluwadare B., Goldberg I.J., Bergman M. (2017). Novel biomarkers for prediabetes, diabetes, and associated complications. Diabetes Metab. Syndr. Obes..

[26] Ortiz-Martínez M., González-González M., Martagón A.J., Hlavinka V., Willson R.C., Rito-Palomares M. (2022). Recent Developments in Biomarkers for Diagnosis and Screening of Type 2 Diabetes Mellitus. Curr. Diab Rep..

[27] Gong X., You L., Li F., Chen Q., Chen C., Zhang X., Zhang X., Xuan W., Sun K., Lao G. (2021). The association of adiponectin with risk of pre-diabetes and diabetes in different subgroups: Cluster analysis of a general population in south China. Endocr. Connect..

[28] Wang Y., Koh W.P., Jensen M.K., Yuan J.M., Pan A. (2019). Plasma Fetuin-A Levels and Risk of Type 2 Diabetes Mellitus in A Chinese Population: A Nested Case-Control Study. Diabetes Metab. J..

[29] Shafaei A., Marjani A., Khoshnia M. (2016). Serum Progranulin Levels in Type 2 Diabetic Patients with Metabolic Syndrome. Rom. J. Intern. Med..

[30] Qu H., Deng H., Hu Z. (2013). Plasma progranulin concentrations are increased in patients with type 2 diabetes and obesity and correlated with insulin resistance. Mediat. Inflamm..

[31] Hassan N.S., Mahran N.A., Hegazy M.G.A. (2020). Assessment of the association of serum progranulin with autophagy in diabetic patients. Endokrynol. Pol..

[32] Tönjes A., Fasshauer M., Kratzsch J., Stumvoll M., Blüher M. (2010). Adipokine pattern in subjects with impaired fasting glucose and impaired glucose tolerance in comparison to normal glucose tolerance and diabetes. PLoS ONE.

[33] Youn B.S., Bang S.I., Klöting N., Park J.W., Lee N., Oh J.E., Pi K.B., Lee T.H., Ruschke K., Fasshauer M. (2009). Serum progranulin concentrations may be associated with macrophage infiltration into omental adipose tissue. Diabetes.

[34] Murakoshi M., Gohda T., Sakuma H., Shibata T., Adachi E., Kishida C., Ichikawa S., Koshida T., Kamei N., Suzuki Y. (2022). Progranulin and Its Receptor Predict Kidney Function Decline in Patients With Type 2 Diabetes. Front. Endocrinol..

[35] Nikolova D., Kamenov Z., Hristova J., Gateva A.T. (2025). Levels of DEFA1, Progranulin, and NRG4 in Patients with Autonomic Neuropathy: Potential Biomarkers for Diagnosis and Prognosis. Metabolites.

[36] Albeltagy E.S., Hammour A.E., Albeltagy S.A. (2019). Potential value of serum Progranulin as a biomarker for the presence and severity of micro vascular complications among Egyptian patients with type 2 diabetes mellitus. J. Diabetes Metab. Disord..

[37] Waluga-Kozlowska E., Kuznik-Trocha K., Komosinska-Vassev K., Olczyk P., Jura-Poltorak A., Winsz-Szczotka K., Telega A., Ivanova D., Strzoda W., Zimmermann A. (2021). Progranulin and chemerin plasma level in obese patients with type 2 diabetes treated with a long-acting insulin analogue and premixed insulin analogue. J. Physiol. Pharmacol..

